# Challenges in monitoring GnRH analog treatment in central precocious puberty

**DOI:** 10.20945/2359-3997000000241

**Published:** 2020-04-27

**Authors:** Ana Claudia Latronico

**Affiliations:** 1 Unidade de Desenvolvimento e Laboratório de Hormônios e Genética Molecular LIM-42 Faculdade de Medicina Universidade de São Paulo São Paulo SP Brasil Unidade de Desenvolvimento e Laboratório de Hormônios e Genética Molecular LIM-42 , Disciplina de Endocrinologia e Metabologia, Faculdade de Medicina da Universidade de São Paulo , São Paulo , SP , Brasil

Central precocious puberty (CPP) results from an early activation of the hypothalamic-pituitary-gonadal (HPG) axis (1,2). The causes of CPP are heterogeneous, with central nervous system abnormalities being of special interest in boys, while idiopathic etiology is more prevalent among girls (3). In the last decade, however, the number of idiopathic cases has diminished thanks to the discovery of mutations in different genes, including *KISS1, KISS1R, MKRN3* , and *DLK1* (2,3).

GnRH analogs (GnRHa) are the mainstay of treatment for this endocrine pediatric condition (4,5). These drugs act by causing a transient downregulation and suppression of the HPG axis. An impressive expansion in the amount of extended-release GnRHa formulations has occurred in the last years. These include leuprolide acetate 3-month depot, triptorelin 3-month depot, triptorelin 6-month depot, and histrelin subcutaneous implant replaced annually (5). These forms are reported to be safe and provide sustained gonadotropin suppression, slowing puberty progression and improving predicted adult height. Notably, the treatment with GnRHa has been considered successful if pubertal progression is halted and if growth velocity and the rate of skeletal maturation are slowed (3,4). However, there are no homogeneous consensus regarding the optimal strategy for monitoring treatment in CPP beyond auxological parameters and periodic bone age X-rays. The HPG axis can be evaluated by measuring unstimulated or stimulated (following GnRH or GnRHa administration) serum LH and sex steroids. It is known that unstimulated LH concentrations above the prepubertal range do not necessarily indicate a lack of suppression, while concentrations within the prepubertal range likely indicate suppression (4). GnRHa stimulation test should be performed for definitive information. However, the lack of correlation between biochemical measurements during treatment and adult height outcomes does not support routine biochemical testing in all patients (4). Therefore, the assessment and management of CPP remain challenging for pediatric endocrinologists.

In a prospective study published in this issue of *AE&M* , Yüce O and cols. (6) showed that first-voided urinary LH measurement was useful to assess pubertal suppression throughout GnRHa treatment in children with CPP. Serum and urinary LH levels were measured using electrochemiluminescence assay (ECLIA) in this new study. The authors demonstrated that urinary LH levels were significantly different among patients with adequate and inadequate hormonal suppression in response to GnRHa treatment. Although the number of studied subjects was small, 69 patients completed the study and only 12 composed the group with inadequate hormonal suppression. This Turkish study showed that urinary LH levels were at least as sensitive as traditional GnRH stimulation and/or GnRHa tests in monitoring treatment. In addition, they suggested a cutoff value with high sensitivity and specificity. It is possible that this first-voided urinary measurement reflects nocturnal excretion of LH, with potential detection of CPP at an early phase. These clinical and laboratorial findings suggest that urinary gonadotropin measurement can be an alternative approach for assessing the hormonal suppression under GnRHa treatment in children with CPP.
